# Efficacy of different neoadjuvant treatment regimens in BRCA-mutated triple negative breast cancer: a systematic review and meta-analysis

**DOI:** 10.1186/s13053-022-00242-0

**Published:** 2022-09-09

**Authors:** Olga Caramelo, Cristina Silva, Francisco Caramelo, Cristina Frutuoso, Leonor Pinto, Teresa Almeida-Santos

**Affiliations:** 1 Gynecology Department, Coimbra Hospital and University Centre (CHUC), EPE, Praceta Prof. Mota Pinto, 3000-075 Coimbra, Portugal; 2 Faculty of Pharmacy of the University of Coimbra, Rua Filipe Simões n° 33, 3000-186 Coimbra, Portugal; 3 Laboratory of Biostatistics and Medical Informatics, iCBR – Faculty of Medicine, University of Coimbra, 3000-354 Coimbra, Portugal; 4 Oncology Department, Coimbra Hospital and University Centre (CHUC), EPE, Praceta Prof. Mota Pinto, 3000-075 Coimbra, Portugal; 5 Centre for Fertility Preservation, Human Reproduction Department, Coimbra Hospital and University Centre (CHUC), EPE, Praceta Prof. Mota Pinto, 3000-075 Coimbra, Portugal; 6 Faculty of Medicine of the University of Coimbra, Azinhaga de Santa Comba - Celas, 3000-548 Coimbra, Portugal

**Keywords:** Triple negative breast cancer, BRCA, Neoadjuvant chemotherapy, Cisplatin, Carboplatin, PARPi

## Abstract

**Purpose:**

Triple negative breast cancer (TNBC) is an aggressive breast cancer strongly associated with *BRCA* mutation. Standard neoadjuvant chemotherapy remains the standard of care for early stage TNBC, the optimal chemotherapy regimen is still a matter of discussion. Other agents, such as poly-ADP-ribosyl polymerase inhibitors (PARPi) and anti-vascular endothelial growth factor (VEGF) antibodies were evaluated in the neoadjuvant setting. This systematic review and meta-analysis intend to evaluate the impact of neoadjuvant treatments in pCR rates in TNBC g*BRCA* mutation, beyond traditional standard chemotherapy.

**Methods:**

PubMed, Clinicaltrials.gov, Cochrane CENTRAL, Embase and key oncological meetings for trials were searched for studies reporting neoadjuvant chemo-immunotherapy in *BRCA* positive TNBC.

**Results:**

Out of 1238 records reviewed, thirty-one trials were included, resulting in a total 619 BRCA-mutated TNBC patients. In *BRCA* mutated TNBC patients who received cisplatin in monotherapy the proportion of patients who achieved pCR was 0.53 (95%CI [0.30, 0.76]), and when treatment combined standard chemotherapy and platin derivatives the proportion of pCR increased to 0.62 (95% CI [0.48, 0.76]). The group of patients treated with platin derivatives, anthracyclines ± taxanes achieved the highest proportion of pCR, 0.66. Patients treated with PARPi alone show a pCR proportion of 0.55 (95% CI [0.30, 0.81]); and when standard chemotherapy and platin derivatives were combined with PARPi the proportion of pCR did not vary.

**Conclusions:**

Patients with *BRCA* mutated TNBC treated with cisplatin in monotherapy demonstrate inferior proportion in the pCR achievement when compared with standard chemotherapy plus platin derivates. The best pCR was achieved with platin derivates in association with anthracyclines ± taxanes. No difference in pCR was found between PARPi alone vs PARPi with standard chemotherapy.

## Introduction

Triple-negative breast cancer (TNBC) accounts for approximately 15% of all breast cancers and represents a great clinical challenge in the clinic, since it is associated with a larger rate of recurrence and a poorer survival [1]. TNBC is characterized by the absence of hormonal receptors and no amplification of human epidermal growth factor receptor-2 (HER2) gene [2].In contrast with other subtypes, systemic treatments for early TNBC have been restricted to traditional chemotherapy regimens for decades.

For patients with early stage TNBC, the use of NACT has become a standard approach [3], despite its impact on the long-term outcomes being controversial [4]. The main aims of neoadjuvant chemotherapy (NACT) are to reduce the extent of surgery, to attain the good prognostic impact of pathologic complete response (pCR) and to guide adjuvant therapy according to the response. Approximately 30–40% of all TNBC patients achieve a pCR after standard neoadjuvant regimens including anthracycline, taxane and cyclophosphamide [2, 5]. TNBC patients who achieve pCR after NACT have shown a significantly reduced risk of relapse and death, compared with patients with residual disease – consequently, it is widely accepted that achieving pCR has a strong favorable prognostic value [6]. Moreover, pCR is associated with lower rates of systemic and local recurrence, as well as a predictor of excellent survival regardless of tumor subtype [6, 7]. Hence, optimization of NACT regimens with the aim of increasing pCR rates has been considered a promising approach for improving prognosis in TNBC.

Approximately 75% of breast cancers containing germline mutations in *BRCA* genes (g*BRCA*) show a triple negative phenotype, with *BRCA*1 dysfunction frequently being one of the main drivers [8]. Among all of patients with TNBC, 10–15% of patients have g*BRCA* mutations [9]. Breast cancers with germline *BRCA1* or *BRCA2* pathogenic or likely pathogenic variants and biallelic inactivation show evidence of deficiency in homologous recombination repair [10, 11].

The loss of BRCA function may turn these tumors particularly sensitive to DNA damaging agents, including platinum agents and poly [ADP- ribose] polymerase inhibitors (PARPi). In patients with g*BRCA* mutations, PARPi have proved to be an effective treatment option in the metastatic setting [12–14] and are currently they are being explored in the early setting of the disease [15]. Platinum agents (i.e. carboplatin and cisplatin) are cytotoxic DNA damaging compounds leading to DNA strand breaks; this mechanism of action is especially active in cancer cells with DNA repair deficiency such as those harboring deleterious mutations in *BRCA* genes. In TNBC patients, platinum-based NACT is associated with significantly increased pCR rate [16]. These agents have shown activity in cancers with g*BRCA* mutation, as BRCA 1/2 proteins have an essential role at repairing the DNA damage [17, 18]. However, the efforts to select a clinically or biologically defined subgroup of patients, who will benefit from the addition of carboplatin, have to date not been conclusive [19]. Several trials demonstrated the effectiveness in the preoperative setting of platinum-based chemotherapy for TNBC patients with g*BRCA* mutations [18]; although, two randomized clinical studies showed that the addition of platinum to standard neoadjuvant chemotherapy significantly increased pCR rate in TNBC regardless of the presence of *BRCA* mutation [16]. Nevertheless, BRCA status is considered a predictive factor of response to chemotherapy leading to higher pCR rates and better disease-free survival in the neoadjuvant setting [20–22].

Bevacizumab is a humanized monoclonal antibody that targets the main isoforms of circulating vascular endothelial growth factor (VEGF), resulting in the inhibition of angiogenesis, cell tumor growth, and cell survival. Bevacizumab use has been investigated in both advanced and early-stage breast cancer treatments, showing an increased response rate, mainly in TNBC patients [23]. The treatment of g*BRCA* mutated breast cancer patients through the use of directed agents for that patient subset is an active area of research.

Since only one third of patients responds to chemotherapy, the identification of novel molecular drivers is crucial for the development of effective targeted treatments. Recently, several clinical trials researching beyond conventional cytotoxic agents showed promising results [24].

To improve the outcome of patients with g*BRCA* mutated TNBC, several approaches for increasing the efficacy of NACT have been pursued. This systematic review and meta-analysis intend to evaluate the impact of different neoadjuvant treatments in pCR rates in this population, beyond traditional standard chemotherapy.

## Methods

We performed the present systematic review according to the PRISMA (Preferred Reporting Items for Systematic Reviews and Meta-Analyses) guidelines [25]. The Prospero registration number is CRD42020192946.

### Search strategy and selection criteria

The following electronic bibliographic databases were systematically searched: MEDLINE, Web of Science database, Embase and Cochrane CENTRAL. All clinical trials regarding NACT in early *BRCA-*mutated TNBC that were published from 2001 to 2021 were retrieved, with no language restriction. Abstracts and presentations from the American Society of Clinical Oncology (ASCO), the European Society for Medical Oncology (ESMO) and the San Antonio Breast Cancer Symposium (SABCS) from 2001 to 2021, were also reviewed to identify relevant unpublished studies.

Two investigators (OC and CS) independently searched the databases, using search terms mainly relating to neoadjuvant treatment in *BRCA* TNBC patients. Specific keywords and free text terms were combined with Boolean operators. The following search phrase was used: (breast OR mammary) AND (cancer OR cancers OR tumor OR neoplasm OR carcinoma) AND (BRCA) AND (neoadjuvant chemotherapy OR induction chemotherapy OR pre-operative chemotherapy) AND (TNBC OR triple-negative OR triple negative OR basal-like OR HER2 negative) AND (pathological complete response OR pCR), without any limits or restrictions. To be eligible, studies had to meet the following criteria: (1) prospective, retrospective or randomized clinical trial in patients with pathogenic *BRCA* mutated early TNBC; (2) must have enrolled *BRCA* TNBC patients receiving NACT; (3) must have provided data on pCR. We excluded (1) case reports, reviews, meta-analyses, animal, or in vitro studies; (2); ongoing studies with results not presented nor published at the time of the literature search.

The investigators (OC, CS) independently double-screened and reviewed the list of records retrieved in accordance with the above-mentioned criteria, to identify potentially eligible articles. When discrepant opinions on study selection among investigators occurred, a third author functioned as tiebreaker; when no compromise was reached, all authors were consulted.

**Fig. 1:**
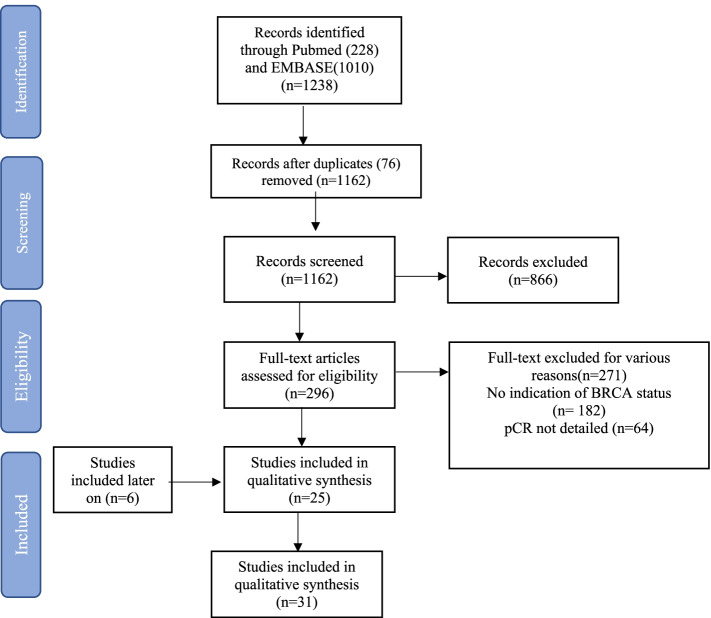
Literature search PRISMA flowchart

The PRISMA flowchart (Fig. 1) summarizes the process of the search strategy for study selection (screening, eligibility, inclusion).

### Data extraction

The following information was extracted and included: study name, first author, year of publication, study design, stage of disease, number of *BRCA* mutated TNBC patients enrolled per regimen(s) of NACT or other treatments, number of patients achieving pCR. Toxicity profile, specifically number of patients with grade 3 or 4 adverse events (AEs) was also retrieved when available. A funnel-plot was performed to assess for potential publication bias -Fig. 10.

### Study objectives

The primary goal of this systematic review and meta-analysis was to compare the efficacy, in terms of pCR of different neoadjuvant treatment regimens other than standard chemotherapy in the population of g*BRCA* mutated TNBC patients.

### Outcomes

The primary efficacy endpoint was the achievement of pCR after neoadjuvant treatment regimens other than standard chemotherapy. The most widely accepted definition of pCR is the absence of residual invasive disease in the breast and sampled axillary nodes (ypT0/is, ypN0). Residual Cancer Burden (RCB) was scored for all patients using the Symmans criteria [26]. Patients who had pCR (RCB 0) were included; near pCR (RCB I) cases were not included in this study.

Other secondary outcomes were evaluated and extracted when available, such as disease-free survival (DFS), overall survival (OS) or event- free survival (EFS).

Regarding safety, the number of patients presenting severe hematological and non-hematological AEs (grades 3 and 4) was retrieved, when reported, for each neoadjuvant treatment regimen.

### Statistical analysis

The authors performed a meta-analysis of the proportion of patients who achieved pCR after treatment with each neoadjuvant regimen. Additionally, a meta-regression was performed adjusting for type of therapy used. The correspondent forest plots were elaborated, and heterogeneity was assessed by the Cochran’s Q test and by statistical coefficient I^2^ of heterogeneity, adopting a statistical significant value of 0.05.

Statistical analyses were conducted using R plataform v3.3.2 with metafor and meta packages [27]. Forest-plots were drawn in MS® Excel®. Relative risk (RR) was calculated by MedCalc Software® v16.1 (https://www.medcalc.org/) to evaluate the impact of the addition of different therapies.

## Results

A total of 1228 studies was identified through the initial search strategy (Fig. 1). After screening the abstracts and reviewing the full texts, a total of 31 trials involving 619 g*BRCA* mutated TNBC patients were selected for the final analysis.

Among the 619 g*BRCA* mutated TNBC patients included in the analysis, 139 patients received cisplatin alone (Table 1). Table 2 shows patients (*n* = 133) who were treated with platin derivatives combined with anthracyclines and taxanes. One single study [28] included 10 patients who were not treated with taxanes, and 18 patients from other study [29]. Fifty-three patients received a combination of standard NACT (anthracycline, cyclophosphamide, taxanes) with carboplatin (Table 3).

**Table 1:** Characteristics of studies included based on treatment regimens: cisplatin in monotherapy

	Affiliation	Type of study	Stage of disease	Nº of B*RCA*1/2 mutated TNBC patients	pCR	Neoadjuvant treatment (Cisplatin 75 mg/m2)
Silver, 2010 [30]	USA	Clinical Trial	II-III	2	2	Cis
Byrski, 2014 [18]	Poland	Clinical Trial	I-III	86	52	Cis
Kolacinka, 2012 [31]	Poland	Clinical Trial	II-III	1	1	Cis
Moiseyenko, 2015 [32]	Russia	Case series	II-III	6	3	Cis
Tung, 2020 [33] ( TBCRC 031)	USA	RCT Phase II	II-III	44	10	Cis

*pCR* Pathological complete response, *Cis* Cisplatin

**Table 2:** Characteristics of studies included based on treatment regimens: platin derivates (cisplatin or carboplatin) and anthracycline with/without taxanes

	Affiliation	Type of study	Stage of disease	Nº of B*RCA*1/2 mutated TNBC patients	pCR	Neoadjuvant treatment
Saether 2018 [28]	Letonia	Retrospective	I-III	10	8	Cis—Dox
Holanek 2019 [29]	Czech Republic	Retrospective	I-III	37	21	Cis- A (18) Cb-P-A (19)
Zhang 2021 [34]	China	Retrospective	I-III	18	9	Cb-A-T
Pohl-Rescigno 2020 GeparOcto [20]	Germany	RCT Phase III	I-III	35	26	Cb-Dox-P
Loib Ann 2018 [22]	15 countries North America Europe Asia–Pacific	RCT Phase III	II-III	33	23	Cb-Dox-P

*pCR* Pathological complete response, *Cis* Cisplatin, *Cb* Carboplatin, *Dox* Doxorubicin, *P* Paclitaxel, *A* Anthracycline based regimen, *T* taxane based regimen

**Table 3:** Characteristics of studies included based on treatment regimens: standard chemotherapy regimen (anthracycline, cyclophosphamide, taxanes) and carboplatin

	Affiliation	Type of study	Stage of disease	Nº of B*RCA*1/2 mutated TNBC patients	pCR	Neoadjuvant treatment
Fontaine 2019 [26]	Belgium	RCT phase II	II-III	9	7	Cb-P + E-C
Sella 2018 [35]	Israel	Clinical trial	I-III	14	9	Cb-P-Dox-C
Walsh 2019 [36]	Irland	Retrospective	I-III	6	4	Cb-P + Dox-C
Loib 2018 BrighTNess [37]	USA	RCT phase III	II-III	24	12	Cb-P- Dox-C

*pCR* Pathological complete response, *Cb* Carboplatin, *Dox* Doxorubicin, *P* Paclitaxel, *E* Epirubicin, *C* Cyclophosphamide

Table 4 presents selected trials with patients treated with carboplatin and taxanes (*n* = 108) and Table 5 present one single study [38] in which patients were treated with carboplatin, iniparib and gemcitabine. Eighty-three patients received standard chemotherapy, carboplatin and a PARPi (Table 6) and 19 patients received a PARPi alone (Table 7). Table 8 describes cases (*n* = 65) who were treated with an anti-VEGF agent associated with standard chemotherapy and carboplatin. Only three patients were treated with eribuline and carboplatin (Table 9).

**Table 4:** Characteristics of studies included based on treatment regimens: carboplatin plus taxanes

	Affiliation	Type of study	Stage of disease	Nº of B*RCA*1/2 mutated TNBC patients	pCR	Neoadjuvant treatment
Gonzalez-Rivera 2016 [39]	Spain	Observational cohort	II-III	13	3	Cb-D
Echvarria 2018 [40]	Spain	non- randomized trial	I-III	9	5	Cb-D
Sharma 2017 PROGECT [41]	USA and Spain	Clinical trial	I-III	27	16	Cb-D
Sharma 2014 [9]	USA	Observational	II-III	14	12	Cb-D
Wunderlee 2018 [42]	Germany	Observational cohort	I-III	15	11	Cb- P
Wang 2015 [43]	China	Observational cohort	I-III	10	4	Cb-P
Menghi 2019 [44]	USA	Phase II Clinical Trial	II-III	9	8	Cb-P
Yuan 2020 [45]	USA	Phase II Clinical Trial	II-III	11	8	Cb-nab-P

*pCR* Pathological complete response, *Cb* Carboplatin, *D* Docetaxel, *P* Paclitaxel, *nab-P* nab-paclitaxel

**Table 5:** Characteristics of studies included based on treatment regimens: carboplatin + gemcitabine + Iniparib

	Affiliation	Type of study	Stage of disease	Nº of B*RCA*1/2 mutated TNBC patients	pCR	Neoadjuvant treatment
Telli 2015 PrECOG 0105 [38]	USA	Phase II Clinical Trial	I-IIIA	16	9	Cb-G-I

*pCR* Pathological complete response, *Cb* Carboplatin, *G* Gemcitabine, *I* Iniparib

**Table 6:** Characteristics of studies included based on treatment regiments: carboplatin + standard NACT + PARPi

	Affiliation	Type of study	Stage of disease	Nº of B*RCA*1/2 mutated TNBC patients	pCR	Neoadjuvant treatment
Severson 2017 [46]	Holand	Multicenter phase II trial	II-III	32	17	Cb-P–V + Dox-C
Loib 2018 BrighTNess [37]	USA	Multicenter, RCT phase III trial	II-III	46	26	Cb-P–V + Dox-C
Litton 2017 [47]	USA	pilot trial,	I-III	5	3	Cb-P–T-Dox-C

*pCR* Pathological complete response, *Cb* Carboplatin, *Dox* Doxorubicin, *C* Cyclophosphamide, *P* Paclitaxel, *V* Veliparib, *T* Talazoparib

**Table 7:** Characteristics of studies included based on treatment regimens: PARPi alone

	Affiliation	Type of study	Stage of disease	Nº of B*RCA*1/2 mutated TNBC patients	pCR	Neoadjuvant treatment
Litton 2020 [15]	USA	Pilot study	I-III	15	7	T
Eikesdal 2019 PETREMAC trial [48]	Norway	Phase II Trial	II-III	4	3	O

*pCR* Pathological complete response, *T* Talazoparib, *O* Olaparib

**Table 8:** Characteristics of studies included based on treatment regiments standard NACT + anti-VEGF antibody

	Affiliation	Type of study	Stage of disease	Median age	Nº of B*RCA*1/2 mutated TNBC patients	pCR	Neoadjuvant treatment
Hahnen 2017 Gepar Sixto [49]	Germany	Phase II RCT	II-III	48	26	17	Cb-P-Dox-Beva
Fasching 2018 Gepar Quinto [21]	Germany	Phase III RCT	I-III	48	39	23	E-C + D-Beva

*pCR* Pathological complete response, *Cb* Carboplatin, *Dox* Doxorubicin, *C* Cyclophosphamide, *P* Paclitaxel, *D* Docetaxel, *E* Epirubicin, *Beva* Bevacizumab

**Table 9:** Characteristics of studies included based on treatment regiments: carboplatin + eribulin

	Affiliation	Type of study	Stage of disease	Median age	Nº of B*RCA*1/2 mutated TNBC patients	pCR	Neoadjuvant treatment
Kaklamani 2015 [50]	USA	Phase II Clinical Trial	II-III	52,5	3	2	Cb-Er

*pCR* Pathological complete response, *Cb* Carboplatin, *Er* Eribulin

### Proportion of pCR achieved

**Fig. 2:**
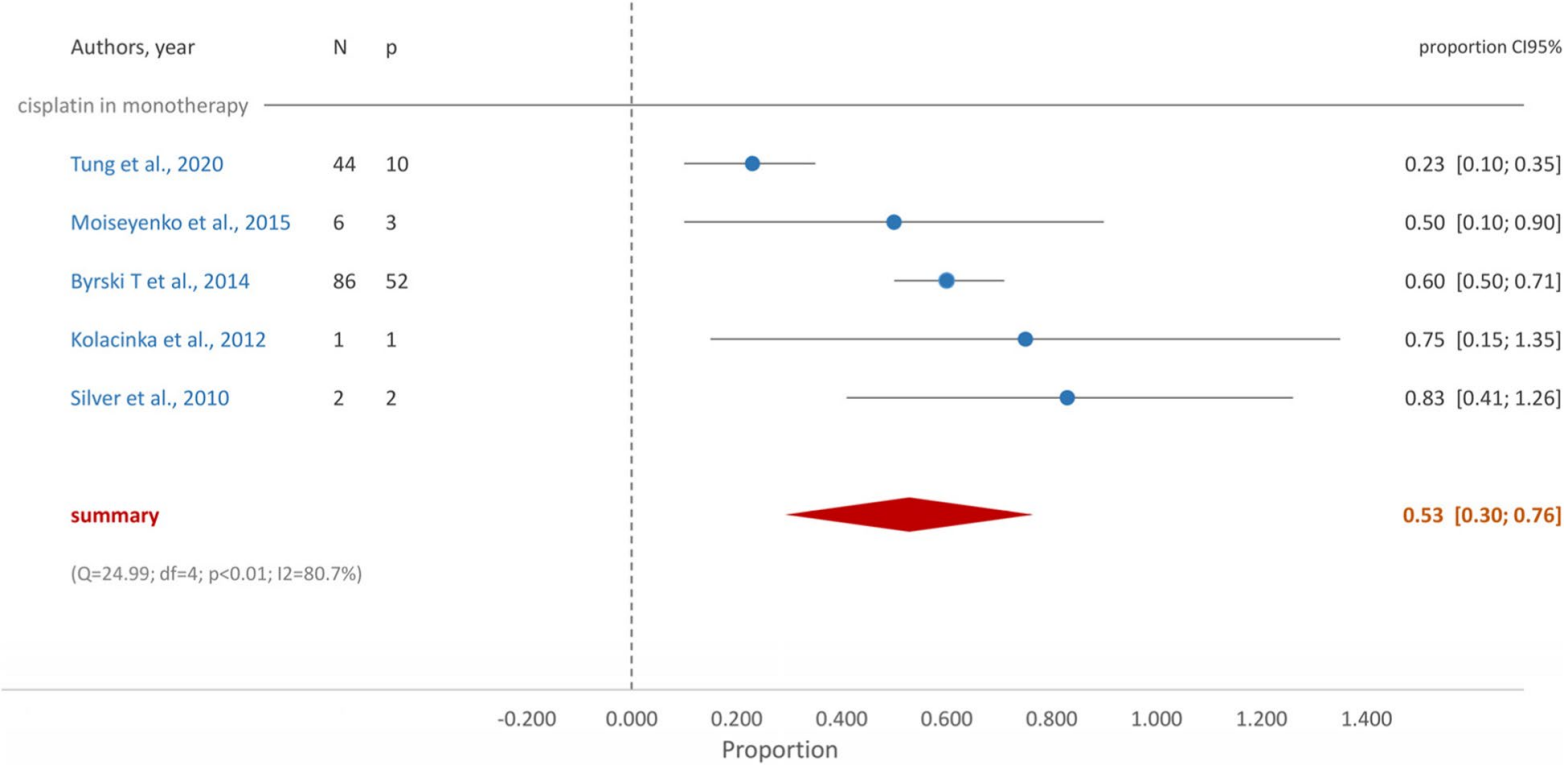
Forest plot- Cisplatin in monotherapy

**Fig. 3:**
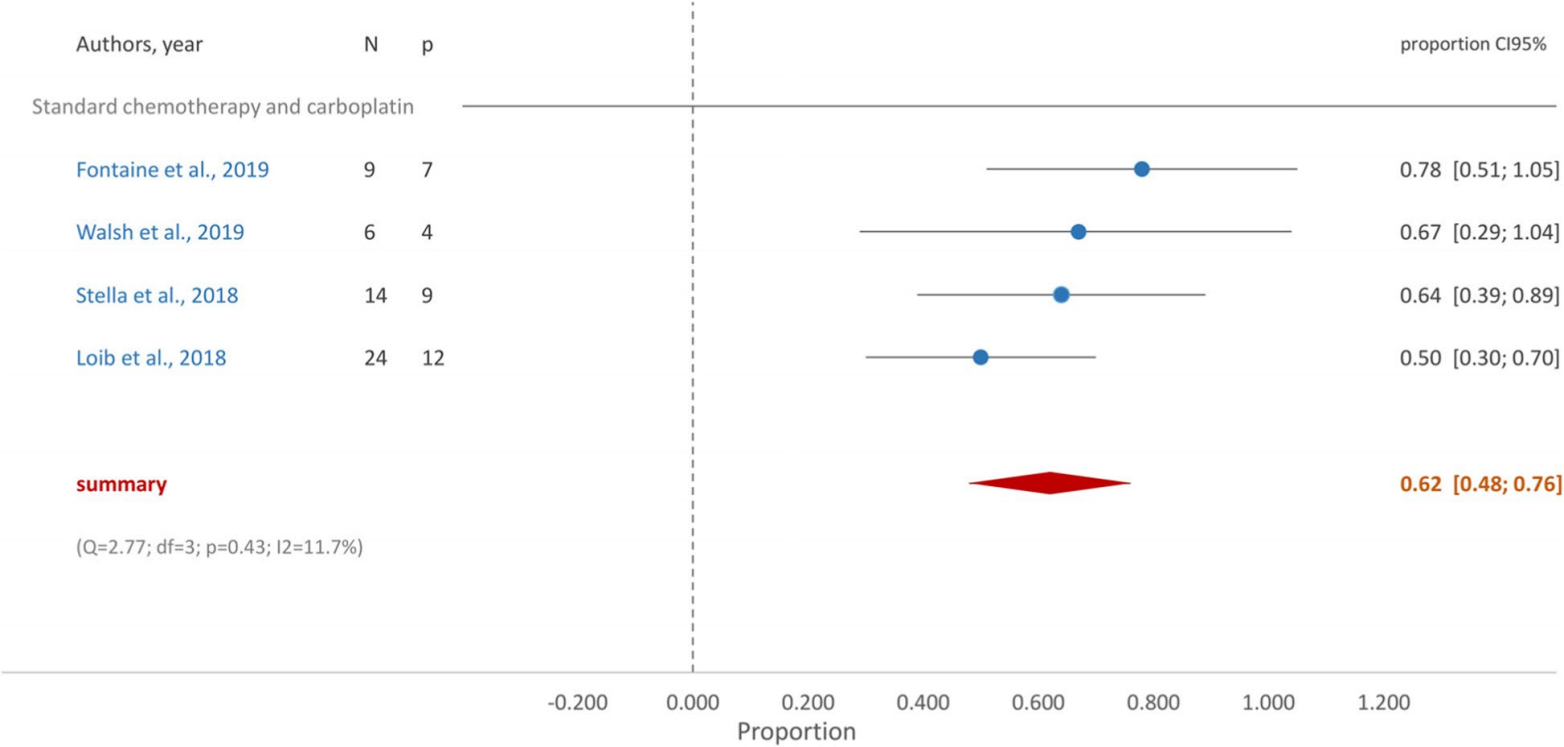
Forest plot- Standard chemotherapy and carboplatin

**Fig. 4:**
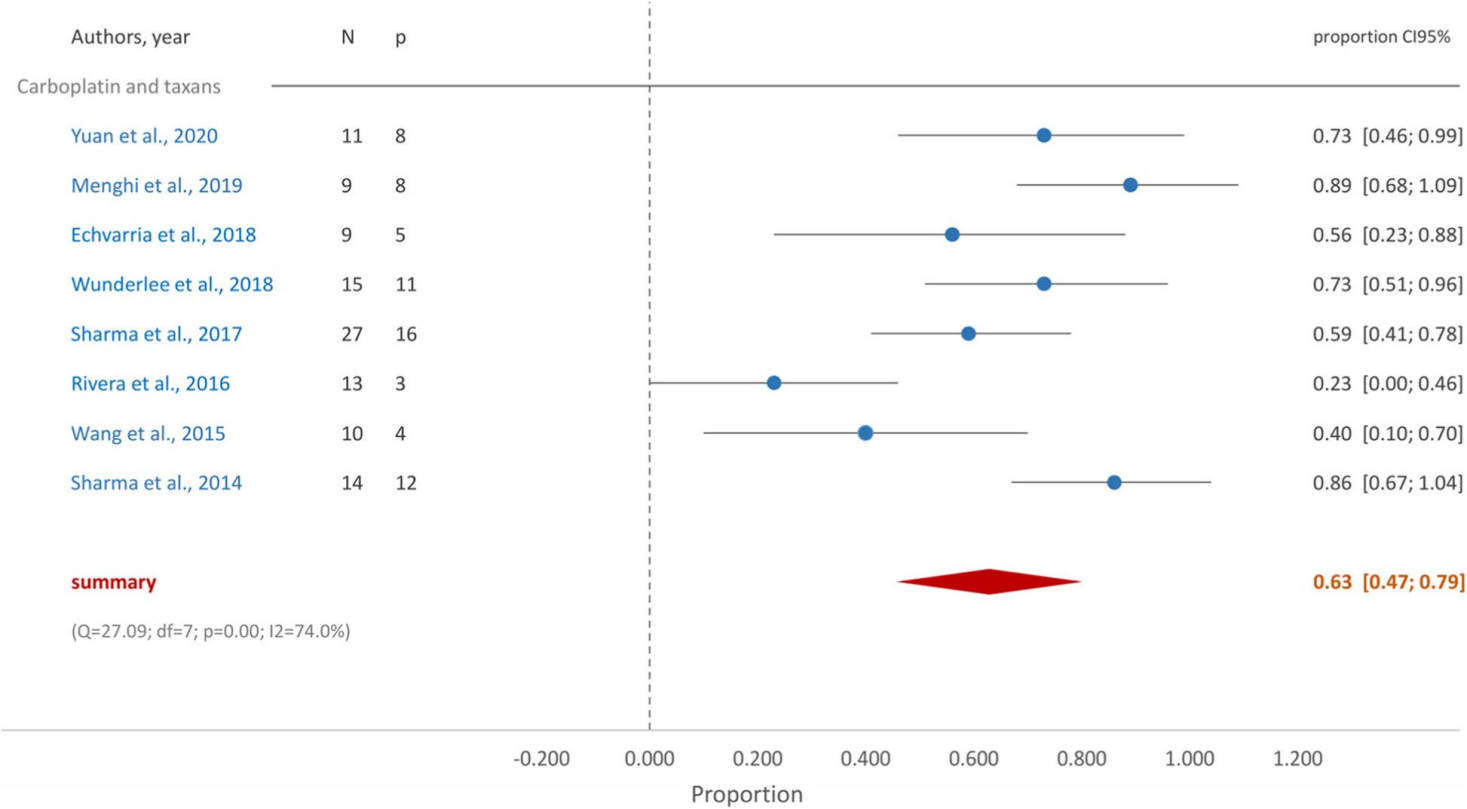
Forest plot- Carboplatin and taxanes

In g*BRCA* mutated TNBC patients who received cisplatin in monotherapy, the proportion of patients who achieved pCR was 0.53 (95%CI [0.30, 0.76]) (Fig. 2). When treatment was a combination of standard chemotherapy and platin derivatives the proportion of pCR increased to 0.62 (95%CI [0.48, 0.76] (Fig. 3). Similarly, the group who received carboplatin and taxane achieved a proportion of pCR of 0.63 (95%CI [0.47, 0.79]) (Fig. 4).

**Fig. 5:**
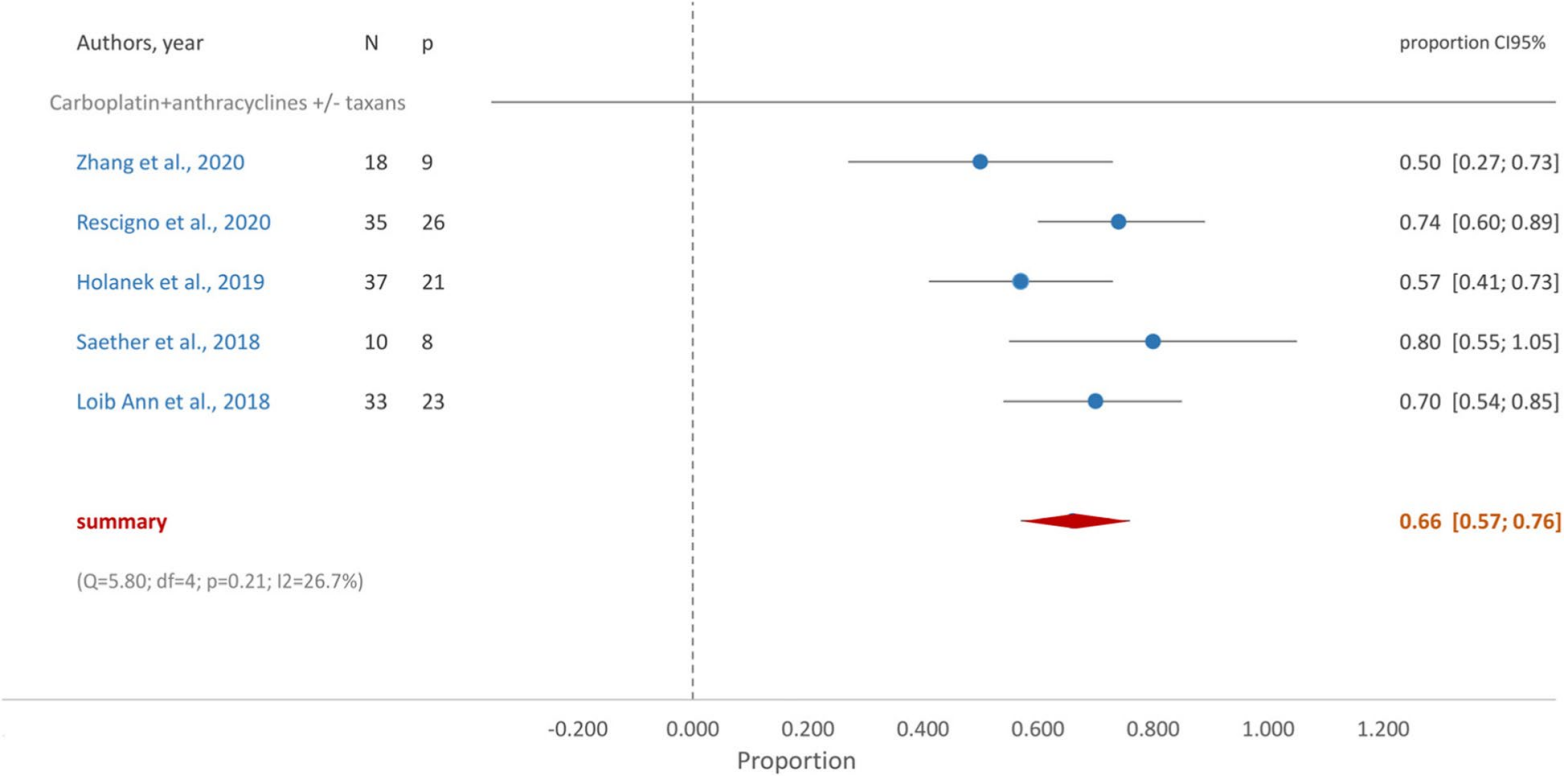
Forest Plot- Carboplatin + anthracyclines ± taxanes

The group of patients treated with platin derivatives, anthracyclines ± taxanes achieved the highest proportion of pCR, 0.66 (95%CI [0.57, 0.76]) (Fig. 5).

**Fig. 6:**
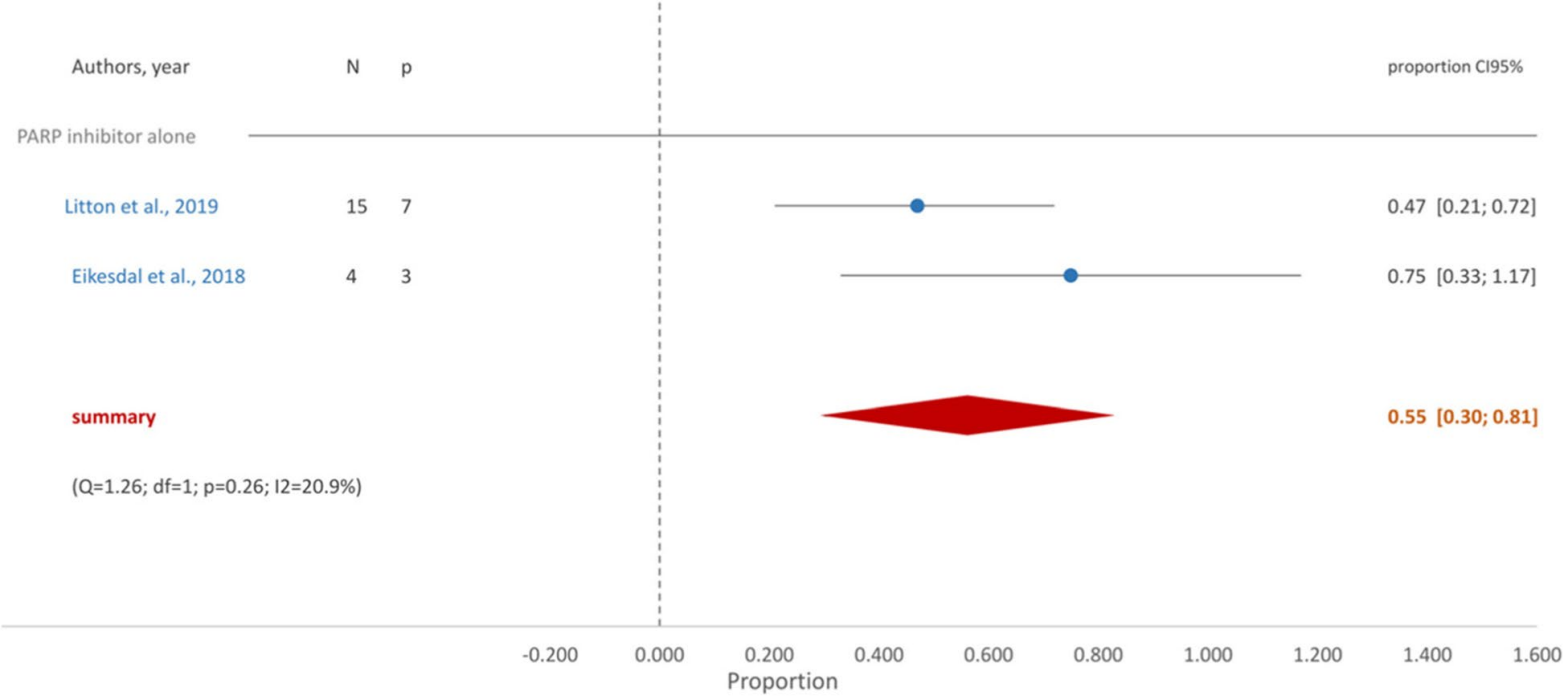
Forest Plot- PARP inhibitor alone

**Fig. 7:**
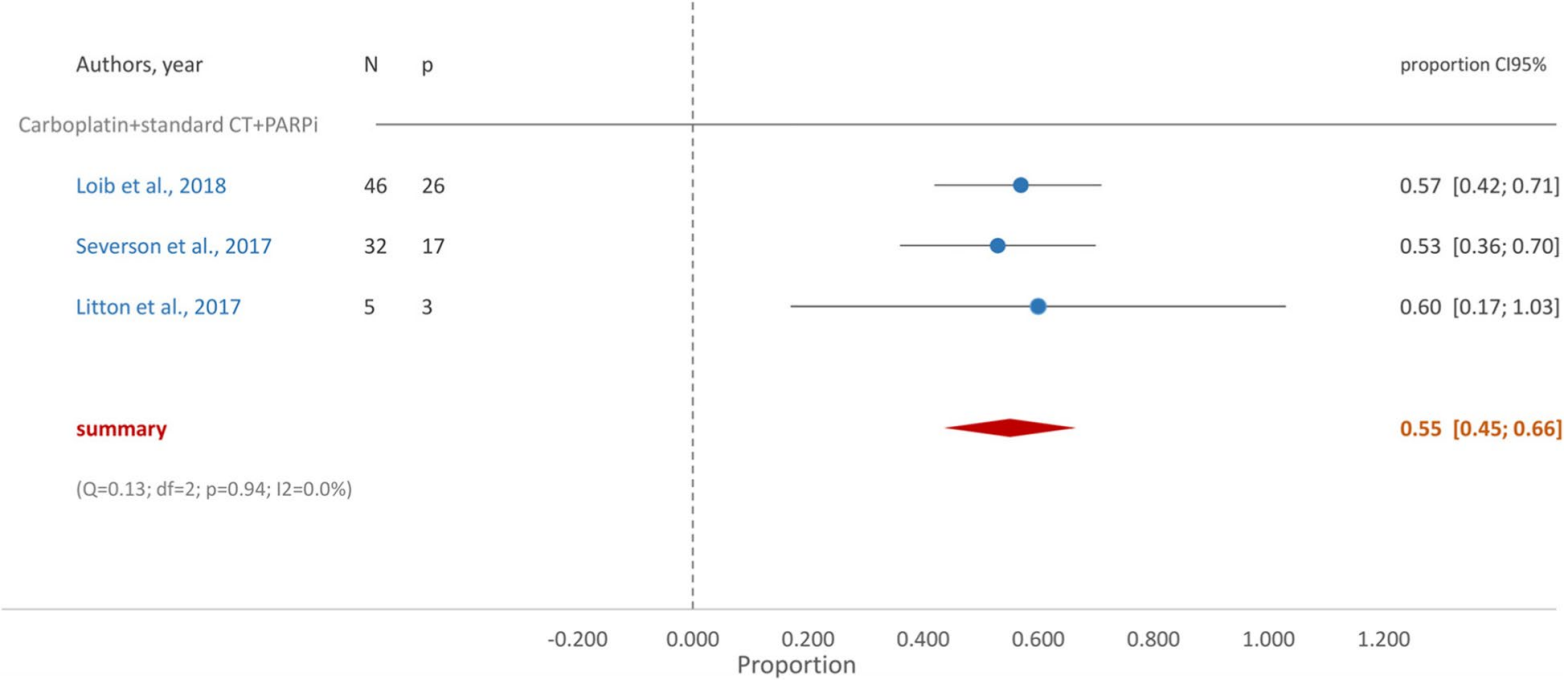
Forest Plot- Carboplatin + Standard NACT + PARPi

With respect to g*BRCA* mutated TNBC patients treated with PARPi alone pCR achievement of was seen in a proportion of 0.55 (95%CI [0.30, 0.81]) (Fig. 6). When standard chemotherapy and platin derivatives were combined with PARPi the proportion of pCR did not change, 0.55 (95%CI [0.45, 0.66]) (Fig. 7).

**Fig. 8:**
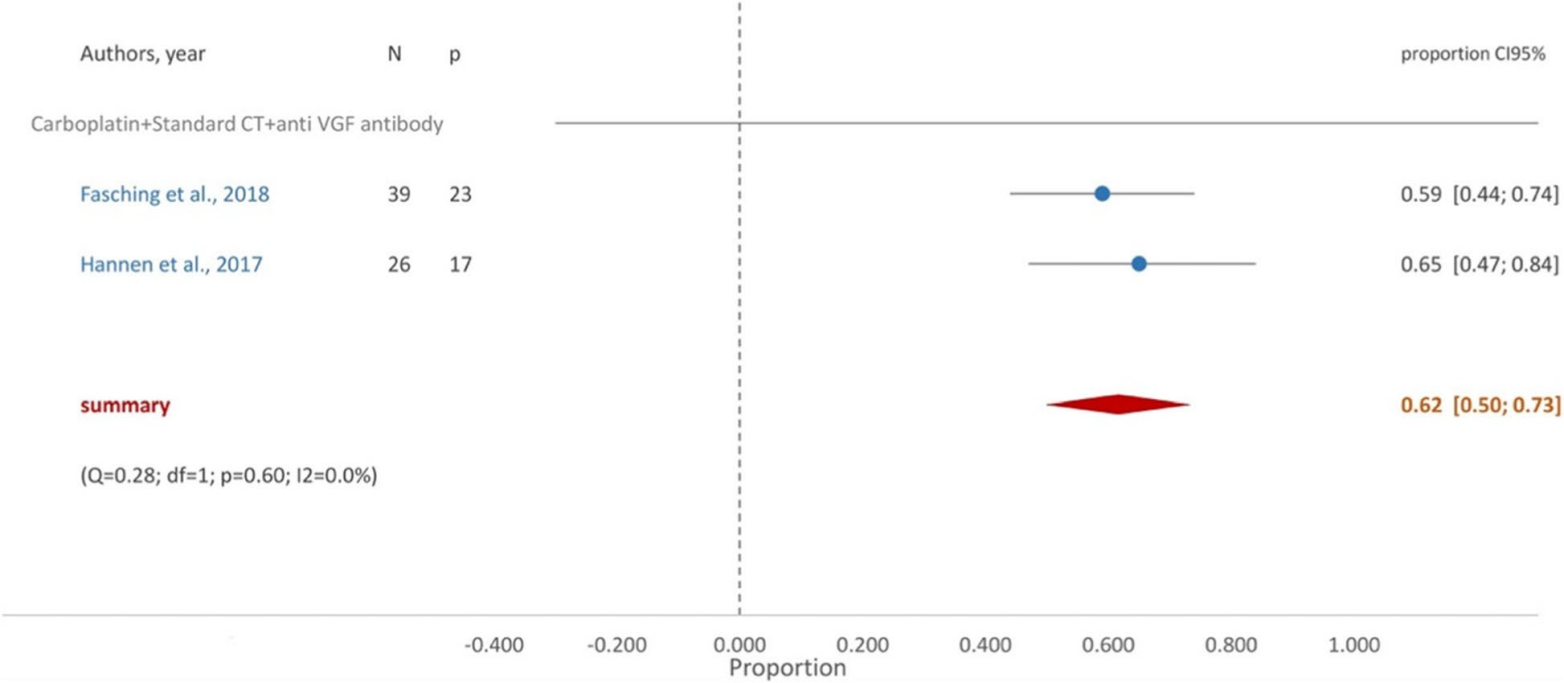
Forest Plot- Carboplatin + Standard NACT + anti VEGF antibody

The group of patients treated with taxane, anthracycline and anti-VEGF (bevacizumab) achieved a proportion of pCR of 0.62 (95%CI [0.50, 0.73]) (Fig. 8) although one study also included carboplatin [49], and another cyclophosphamide in the treatment regimens [21].

Only one study evaluated the association of carboplatin and eribulin, which achieved pCR proportion in two-thirds of patients [50].

**Fig. 9:**
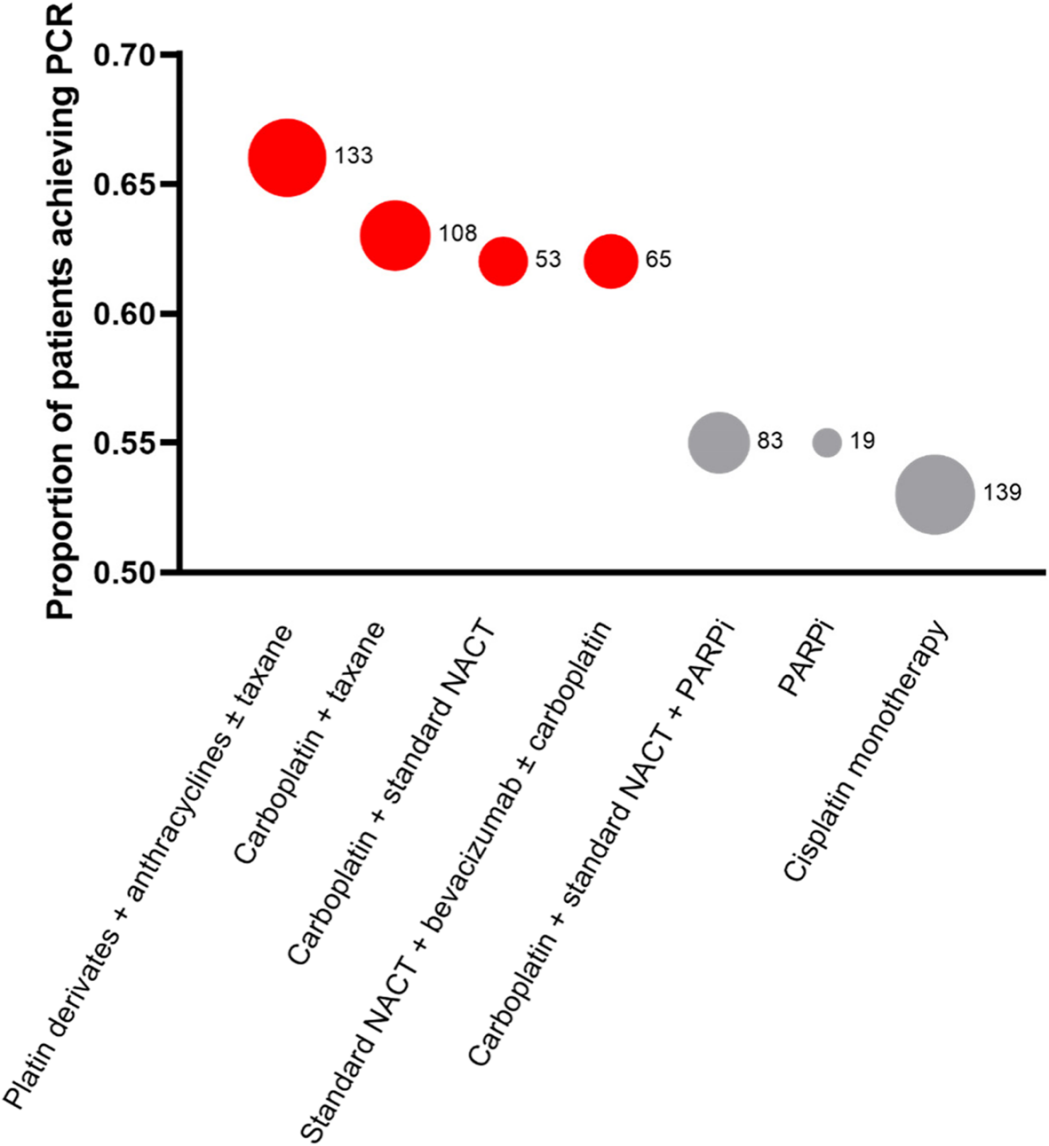
Bubbleplot graphic showing the proportion of different treatments in pCR achievement. The size of a bubble represents the number of patients included in each group

Figure 9 displays the proportion of pCR achieved with different treatment regimens and the corresponding number of patients. The largest group was treated with cisplatin in monotherapy and achieved the lowest proportion of pCR achievement. On the other hand, the highest pCR was achieved in the group treated with platin derivates, anthracyclines ± taxanes which included the second highest number of patients.

**Fig. 10:**
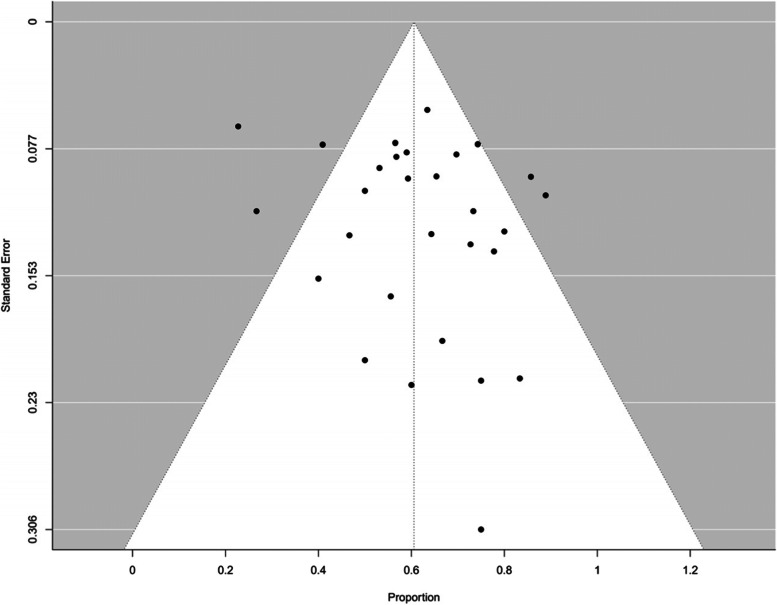
Funnel plot assessment of publication bias for pCR in patients receiving NACT and platinum-based therapies for early TNBC with g*BRCA* mutations

The symmetric funnel plot for this meta-analysis shows an additional indicator of the absence of publication bias and study heterogeneity (Fig. 10).

### Effect of specific treatment agents on pCR achievement

#### Addition of taxanes to platin derivatives

The effect of platin derivatives in pCR achievement significantly increased when a taxane was added (Relative Risk (RR), RR = 1.268; 95% CI [1,013, 1.588]), indicating a positive effect of combining carboplatin and taxanes.

#### Addition of standard chemotherapy to platin derivates

The effect of standard NACT in achieving pCR in g*BRCA* mutated TNBC patients was evaluated by comparing standard NACT with platin derivates versus platin derivates alone, showing a RR of 1.234 (95% CI [0.936, 1.672]).

#### Addition of PARPi to standard NACT

The effect of PARPi in achieving pCR in gBRCA mutated TNBC was evaluated by comparing the addition of PARPi to standard NACT with platin derivates versus standard NACT with platin. A RR of 1,089 95% CI [0.814, 1.458]) was found, denoting no contribution of PARPi in this setting.

The impact of standard NACT plus platin in the presence of PARPi versus PARPi alone was also assessed, resulting in a RR of 1,053 95% CI [0.659, 1.681]).

#### Addition of anti-VEGF

The combination of anti-VEGF (bevacizumab) with standard NACT and platin derivates showed a RR of 0.997 (95% CI [0.762, 1.304]) when compared with the same treatment in the absence of anti- VEGF denoting no contribution of anti-VEGF in the achieving pCR.

### Long term outcomes

Long-term outcomes such as DFS, EFS and OS, were reported in some of the included studies but very few discriminate results according to *BRCA* status. Holanek et al. [29] reported 85.5% of DFS after 3 years of follow-up in patients treated with carboplatin, compared with 76.1% of patients who did not receive carboplatin. Fontaine et al. [26] reported EFS an OS as secondary endpoints. From a total of 63 patients, 9 patients relapsed. In Walsh et al. [36], DFS, metastasis-free survival and breast cancer-specific survival were evaluated, with no significant difference between groups. A survival analysis was conducted by Yuan et al. [45] with a DFS of 87.3% and OS of 90.2% for a median follow up of 3-year. Similar results were found by Kaklamani et al. [50] with a median follow-up of 16.8 months and a progression-free survival of 76.8%. Nevertheless, none of the previous studies discriminated the subgroup of TNBC g*BRCA* patients, which might constraint generalization of conclusions.

The longer follow-up (47.3 months) of GeparSixto [49] showed a significant increase in DFS in TNBC when treated with paclitaxel, doxorubicin and carboplatin, but the benefit was restricted to the non-*BRCA* subgroup. In Wunderle et al. [42], patients who achieved pCR had better DFS and OS rates compared with those who did not achieve pCR, regardless of *BRCA1*/*2* mutation status.

Fasching et al. [21] analyzed *BRCA* mutated TNBC patients with regard to prognosis and found that patients with *BRCA* mutations had a significantly better DFS (HR = 0.644, 95% CI[0.415, 0.998], *p* = 0.047) than those with no mutations.

Other long-term outcomes were reported by Zang et al. [34],such as recurrence-free survival (RFS), distant recurrence-free survival and OS, concluding that there were no differences in survival between carriers and non- carriers of *BRCA* mutation who received chemotherapy with platin derivates.

### Safety outcomes

#### Hematological effects

Several studies reported grade 3 and 4 hematological AE (i.e. neutropenia, anemia, and thrombocytopenia) (Table 10). However, most of them did not report adverse effects according to *BRCA* status. The most common hematological adverse effect was neutropenia. This was very significant in patients treated with carboplatin, standard NACT and anti-VEGF agent, with an incidence of 76.6%. On the other hand, groups treated with PARPi or cisplatin alone presented the lowest incidences of neutropenia, 5.8% and 3.6% respectively, while the group treated with carboplatin and eribulin revealed an incidence of 60%. Concerning anemia, the highest incidence was reported in the group treated with carboplatin, standard NACT and PARPi (25.1%).

**Table 10:** Incidence of hematological adverse effects with different neoadjuvant treatment regimens, regardless of *BRCA* status

	**Cisplatin** **alone**	**Carboplatin + anthracycline + taxane**	**Carboplatin + taxane**	**Carboplatin + Gemcitabine + PARPi**	**Carboplatin + NACT + PARPi**	**Carboplatin + NACT + anti-VEGF antibody**	**PARPi**	**Carboplatin + Eribulin**
n	**196**	**106**	**239**	**93**	**398**	**1246**	**52**	**30**
Adverse effect								
Neutropenia	3.6%	50.9%	10.5%	45.2%	59.0%	76.6%	5.8%	60.0%
Anemia	1.0%	20.8%	4.2%	8.6%	25.1%	5.0%	15.4%	23.3%
Febrile neutropenia	0.5%	18.9%	3.3%		1.5%	12.4%		
Thrombocytopenia		12.3%	5.4%	6.5%	13.1%	5.0%	1.9%	20.0%
Leukopenia		12.3%	0.8%		4.5%			

#### Non-hematological effects

Table 11 presents a summary of reported non-hematological AEs. The group of carboplatin, standard NACT and anti-VEGF agent showed the highest incidence of gastrointestinal adverse effects (27,7%) cardiac disorders (6,7%), renal and urinary (4,8%) and skin and subcutaneous tissues AEs (7,1%). The group treated with carboplatin, gemcitabine and PARPi also presented considerable gastrointestinal symptoms, in up to 24,7% of cases.

**Table 11:** Incidence of non-hematological adverse effects with different neoadjuvant treatment regimens

	**Cisplatin** **alone**	**Carboplatin + anthracycline + taxane**	**Carboplatin + taxane**	**Carboplatin + Gemcitabine + PARPi**	**Carboplatin + NACT + PARPi**	**Carboplatin + NACT + anti VEGF antibody**	**PARPi**	**Carboplatin + Eribulin**
n	**196**	**106**	**239**	**93**	**398**	**1246**	**52**	**30**
Adverse effect								
Gastrointestinal disorders	**4.6%**	**2.8%**	**8.4%**	**24.7%**	**6.5%**	**27.7%**	**0.0%**	**0.0%**
Cardiac disorders	**1.5%**	**0.0%**	**0.4%**	**1.1%**	**0.8%**	**6.7%**	**0.0%**	**0.0%**
Nervous system disorders	**0,.0%**	**2.8%**	**1.3%**	**2.2%**	**0.8%**	**1.5%**	**0.0%**	**0.0%**
Renal and urinary disorders	**1,.0%**	**0.0%**	**0.4%**	**1.1%**	**0.0%**	**4.8%**	**0.0%**	**3.3%**
Skin and subcutaneous tissue disorders	**1.0%**	**0.0%**	**0.8%**	**0.0%**	**0.0%**	**7.1%**	**0.0%**	**0.0%**

## Discussion

The goal of this systematic review was to assess the proportion of pCR in g*BRCA* TNBC patients when neoadjuvant treatments regimens other than standard ones were used.

A lot of efforts has been done to identify predictive markers for the use of platinum, driven by the hypothesis that tumors with deficient homologous recombination, such as those with g*BRCA*1/2 mutations, may be better targeted by carboplatin due to their inability to repair double-strand DNA breaks induced by platinum salts. However, its efficacy for breast cancer with *BRCA* germline mutations remains inconclusive.

Our results point to a significant role of standard NACT (anthracyclines, taxanes and cyclophosphamide) in this setting, as the combination of carboplatin with standard regimens yielded a proportion of pCR achievement of 0.62 (95%CI 0.48–0.76), higher than in patients treated with cisplatin alone [0.53 (95%CI 0.30–0.76); risk ratio 1.234 (95% CI 0.936–1.672)].

Accordingly, in our meta-analysis, patients treated with platin derivatives plus an anthracycline with or without a taxane (two studies without taxanes) achieved the highest proportion of pCR, 0.66 (95%CI [0.57, 0.76]), closely followed by the group who received carboplatin and taxane, in which a proportion of pCR of 0.63 (95%CI [0.47, 0.79]) was achieved. Interestingly, analysis of pCR with anthracyclines and taxanes in the presence of platin derivates favored anthracyclines, although hematological AEs increased with these agents.

Previously published meta-analysis that assessed the addition of platinum to standard NACT found an improvement of pCR rates for patients with *BRCA* mutations, although this was not a statistically significant [51,16]. Similar results were obtained in our previous meta-analysis, which revealed an increased pCR rate in *BRCA* mutation carriers (58.4%) compared with non-carriers (50.7%), but with no statistical significance [19].

The lowest proportion of pCR rate in our study (0.53 (95%CI [0.30, 0.76]) was found in the group of 139 patients treated with cisplatin in monotherapy.

Besides platin agents, other neoadjuvant treatments were reported in the trials included in this meta-analysis. PARP enzymes play a major part in DNA repair mechanisms and inhibition of PARP activity leads to the accumulation of double-strand DNA breaks. These breaks are normally repaired by double-strand homologous recombination pathways that include the tumor-suppressor proteins BRCA1 and BRCA2. Thus, g*BRCA* mutated TNBC as well as the *BRCA*ness phenotype are in theory particularly vulnerable to PARPi [52].

In our study, when PARPi (talazoparib or olaparib) were used in monotherapy pCR was the same (0.55 (95% CI [0.30, 0.81])) as when added to standard chemotherapy and carboplatin (0.55 (95% CI [0.54, 0.66])), only with a much lower incidence of hematologic toxicity.

However, when PARPi were added to standard chemotherapy and carboplatin, proportion of pCR was lower than that of treatment with standard chemotherapy and carboplatin in the absence of PARPi (0.62 (95%CI [0.48, 0.76])). These results point to a neutral to non-beneficial effect of PARPi in this setting. This is surprising as it would be expected that the addition of PARPi would increase pCR rate when compared to those treated with standard chemotherapy and carboplatin. This has been shown by a recent study which found significantly longer survival free of invasive or distant disease when using olaparib as adjuvant therapy after neoadjuvant or adjuvant chemotherapy and local therapy in early breast cancer patients with *BRCA1* or *BRCA2* germline pathogenic variant [53]. Likewise, in the I-SPY2 phase 2 trial, the addition of veliparib and carboplatin to standard NACT improved pCR from 26% in the control arm to 51% in the veliparib–carboplatin group of TNBC patients [54].

The VEGF pathway plays a key role in the pathophysiology of TNBC. However, in our study, the addition of bevacizumab to standard chemotherapy with platin derivatives did not yield any benefits since a similar proportion of pCR achievement was obtained in both groups 0.62 (95%CI [0.50–0.73]). Moreover, patients in this group reported the higher incidence of neutropenia (over 76%).

The evaluation of pCR is of extreme importance. However, the real impact of this outcome in long term clinical results is not yet clear. In this study we tried to consider other outcomes but few of the included studies reported long-term outcomes in relation to BRCA status. The vast majority did not discriminate between subgroups and reported outcomes like DFS or OS for the entire group of TNBC patients. Only two studies, GeparSixto [49] and Fasching et al. [21], separately analyzed *BRCA* mutated TNBC patients and found that pCR was a strong predictor of DFS for patients without *BRCA,* but not for patients with *BRCA* mutations. Nevertheless, with regard to prognosis, patients with a *BRCA* pathogenic variant had a significantly better DFS.

It is important to point out that few trials were sufficiently powered enough to assess long-term outcomes in the TNBC g*BRCA* mutated group. Hence, the question of clinical utility of different treatment approaches in this subgroup remains unanswered and further research is necessary.

A recent publication exploring safety issues in the neoadjuvant setting concludes that g*BRCA*1/2 mutated patients show a higher risk of hematological toxicity when treated with regimens including a taxane [55]. On the contrary, our study demonstrates higher adverse hematological AEs with the addition of anthracyclines, PARPi and anti-VEGF to a standard regimen with platin. This difference may be related to the lack of distinction of AEs according to the *BRCA*1/2 status.

Our study presents several limitations. Major limitations are related to the small number of patients with g*BRCA* TNBC included in the different trials and heterogeneity between trials (related to study design, drugs and doses of treatment regimens). Nonetheless, g*BRCA* mutated TNBC patients are rarely distinguished in trials and such approach is considered the only way to obtain conclusions.

Almost 20% of breast cancer patients share histological features and clinical outcomes with *BRCA*1/2 related cancers without detectable gBRCA1/2 mutations, a phenotype defined as *BRCAness.* Beyond g*BRCA* mutations, somatic *BRCA* mutation and *BRCA* silencing through promotor hypermethylation or alterations affecting other genes related to homologous recombination [10] that can mimic the *BRCA*ness state. Importantly, *BRCA1* methylated and g*BRCA1* mutated TNBCs share gene expression and immune profiles and seem to have a similar outcome after adjuvant chemotherapy [56]*.* Consequently, another limitation of our study was to include only *gBRCA* mutations and not all cases with the *BRCA*ness phenotype.

To our knowledge this is the first study that gathers information on g*BRCA* mutation TNBC patients, a subgroup with many singularities often not separately analyzed in published trials. Our assessment of neoadjuvant treatments in this distinct group of TNBC revealed clinically relevant conclusions with possible impact on treatment options. It is also noteworthy that this is the first study in this subset of patients of such a wide range of treatments beyond conventional chemotherapy.

## Conclusions

This study showed that patients with g*BRCA* mutated TNBC patients treated with cisplatin in monotherapy in the neoadjuvant setting present a lower pCR when compared with standard chemotherapy combined with platin derivatives, strengthening the role of standard chemotherapy. Likewise, the addition of PARPi to standard chemotherapy and carboplatin decreased the proportion of pCR denoting no contribution of PARPi in this setting and favoring the role of standard chemotherapy and platin derivates. The highest proportion of pCR was found with the combination of platin derivates and anthracyclines ± taxanes.

## Data Availability

Not applicable.
